# Acute Effects of Elastic Bands as Resistance or Assistance on EMG, Kinetics, and Kinematics During Deadlift in Resistance-Trained Men

**DOI:** 10.3389/fspor.2020.598284

**Published:** 2020-11-05

**Authors:** Vidar Andersen, Helene Pedersen, Marius Steiro Fimland, Matthew Peter Shaw, Tom Erik Jorung Solstad, Nicolay Stien, Kristoffer Toldnes Cumming, Atle Hole Saeterbakken

**Affiliations:** ^1^Faculty of Education, Arts and Sports, Western Norway University of Applied Sciences, Sogndal, Norway; ^2^Department of Neuromedicine and Movement Science, Faculty of Medicine and Health Sciences, Norwegian University of Science and Technology, Trondheim, Norway; ^3^Unicare Helsefort Rehabilitation Center, Rissa, Norway; ^4^Faculty of Health and Welfare, Østfold University College, Halden, Norway

**Keywords:** velocity, force, variable resistance, hip extension, gluteus maximus, hamstring, erector spinae

## Abstract

The aim of the study was to compare neuromuscular activation, kinetics and kinematics in three variations of the deadlift: (1) free weights, (2) free weights with elastic bands as resistance (bands anchored to the ground) and (3) free weights with elastic bands as assistance (bands attached above the bar). Sixteen resistance-trained men performed one repetition of the three variations as fast as possible using a 2-repetition maximum load in randomized and counterbalanced order. Muscle activation (gluteus maximus, semitendinosus, biceps femoris, erector spinae, vastus lateralis, and vastus medialis), kinematics (average-, peak-, and time to peak velocity), and kinetics (average-, peak,-and time to peak force) were measured during the ascending movement. Resisted and assisted deadlifts led to higher average and peak force outputs (*p* < 0.001–0.037, ES = 0.29–0.58), and time to peak velocity was shorter when compared to the free weights deadlift (*p* = 0.005–0.010, ES = 0.83–1.01). However, peak force was achieved faster when using free weights (*p* < 0.001, ES = 1.58–2.10) and assisted deadlifts had a lower peak velocity compared to resisted and free weights deadlift (*p* = 0.004–0.046, ES = 0.43–0.60). There were no significant differences in muscle activation between the different conditions (*p* = 0.082–1.000). In conclusion, the assisted and resisted deadlift produced higher force when compared to free weights. However, free weight and resisted deadlift seem more favorable for the barbell velocity. These findings are of importance for athletes and coaches which should select exercise depending on the goal of the session.

## Introduction

Deadlift is a popular exercise among athletes and recreational lifters seeking to increase muscle hypertrophy and maximum and explosive strength (Kompf and Arandjelovic, 2017). When performing an exercise with free weights, the maximal load lifted is often dictated by a short section within the range of motion (ROM) called the sticking region (van den Tillaar et al., 2014). Beyond the sticking region there will be a mismatch between the external torque and the potential of the muscular torque (Gabriel et al., 2006; Frost et al., 2010). That is, when you have crossed the sticking region, the lift becomes quite easy. In exercises with an ascending force curve such as deadlift, squat, and bench press, combining free weights and elastic bands have been proposed as an alternative to reduce this mismatch and therefore optimize the relationship between muscle- and free weight torque (Frost et al., 2010; Wallace et al., 2018). The elastic bands induce a variable resistance as they are stretched due to their elasticity (McMaster et al., 2010), eliciting increasing muscular demand throughout the ROM.

The use of elastic bands, as a means of providing variable resistance, can be implemented in two ways; resistance and assistance (McMaster et al., 2009; Argus et al., 2011). When using the elastic bands for additional resistance, bands are anchored from the floor and attached to the barbell, and requires the lifter to perform with less load on the barbell than when performing without bands. Whereas, elastic bands that are used for assistance are attached from a position that is above the barbell, allowing an individual to lift a greater barbell load.

Two previous studies have compared the kinetics and neuromuscular activation during free weight deadlift and resisted deadlift using elastic bands (Galpin et al., 2015; Heelas et al., 2019). Both studies found that combining free weights and elastic bands increased the velocity in the lift when compared to only free weights. Furthermore, Galpin et al. (2015) found the rate of force development (RFD) to be higher in the resisted deadlift when lifting at a high intensity [85% of 1-repetition maximum (RM)]. However, the free weight condition appeared favorable for both average and peak force. Heelas et al. (2019) found similar mean activation between the free weight- and band conditions, although free weights led to a higher peak activation in the semitendinosus and medial gastrocnemius. Importantly, both studies matched the load in the upper position i.e., the band and free weight-conditions developed the same absolute load in the top position. It could be argued that matching the load based on relative intensity (i.e., the same RM) would be more specific toward training (McBride et al., 2010). Swinton et al. (2011) used chains instead of elastic bands when they compared deadlift using constant or variable resistance. In contrast to Galpin et al. they found the variable resistance to increase the force, but reduce the velocity when compared to free weights. Finally, only one study has compared free weight deadlift with assisted deadlift (Andersen et al., 2019). Andersen et al. (2019) compared muscle activation in the hamstring, gluteus, and erector spinae muscles between 2 RM in free weights vs. free weights in combination with either a high or a low contribution from elastic bands among resistance-trained men. The results showed a higher activation in the erector spinae, favoring the high assistance condition when compared to the low assisted condition, but with no difference to the free weights.

Previous studies examining the properties of elastic bands have shown that the resistance will not increase linearly when the elastic bands are lengthened, but instead increase in a more curvilinear tension-deformation relationship (McMaster et al., 2010; Shoepe et al., 2010). Therefore, there is a potential for higher resistance throughout the ROM performing assisted deadlifts compared to resisted deadlifts. This knowledge could be of important value for athletes and coaches when designing their resistance-training program. To the authors‘ knowledge, there have been no previous studies examining the acute effects between free weights, assisted and resisted deadlifts on neuromuscular activation and kinetics when using the same relative intensity.

Therefore, the aim of the study was to compare neuromuscular activation, kinetics and kinematics between free weight-, resisted- and assisted deadlift using a matched relative intensity (one rep using 2-RM loading). Based on the previous study using relative intensity (Andersen et al., 2019) we hypothesized that the lower back would demonstrate higher activation during the elastic band-conditions compared to the free weights. Further, we hypothesized both band conditions to produce more force than the free weights, but the assisted band-conditions to be favorable among the two. Finally, we hypothesized the resisted deadlift to produce the highest velocity.

## Materials and Methods

### Experimental Approach to the Problem

**Figure 1:**
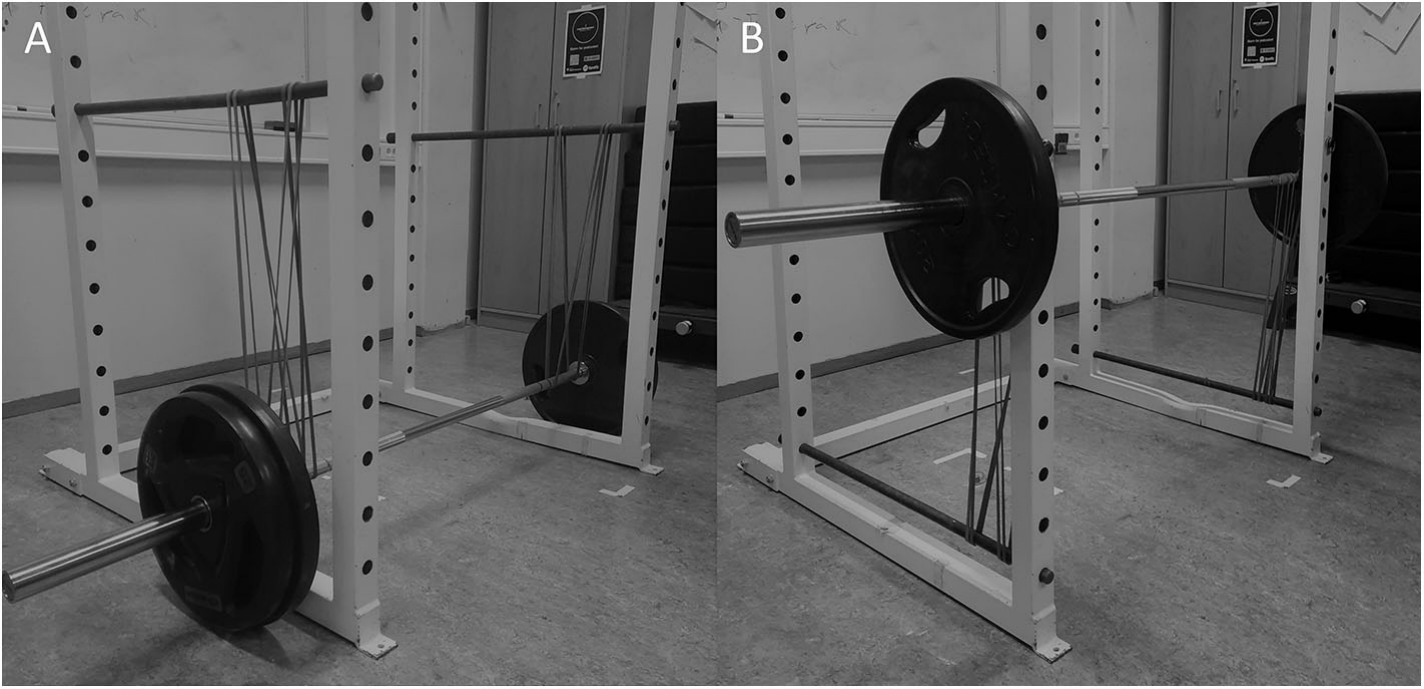
Illustration of the set-up, showing lower position in the assisted **(A)** and top position in the resisted **(B)** deadlift. Length of the elastic bands are equal in the two positions.

To compare the effects of the three different conditions on neuromuscular activation, kinematics and kinetics, a within-subjects, repeated measures design was used. After three familiarization sessions determining the 2-RM in each condition, the participants performed one repetition (only ascending phase) in the conventional free weight deadlift, assisted deadlift and resisted deadlift as fast as they could, using 2-RM loads. The order of the exercises was randomized and counterbalanced. In the assisted-condition (Figure 1A), the elastic bands provided progressively less load/assistance to the barbell throughout the ascending lift, consequently demanding more plates on the barbell when compared to the free weight-condition. In the resisted-condition (Figure 1B), the elastic bands act in an opposite manner, progressively loading the barbell as the barbell is lifted, hence, requiring less plates compared to the free weight-condition. Muscle activation (gluteus maximus, semitendinosus, biceps femoris, erector spinae, vastus lateralis and vastus medialis), kinematics (average velocity, peak velocity, time to peak velocity) and kinetics (average force, peak force and time to peak force) were measured during the ascending part of the lift. If any significant differences were found in EMG, average force or average velocity between the different conditions, the whole movement was divided into a lower and upper phase, to better understand the pattern of the variables during the lift. The classification of each phase was made from the trajectory of the barbell, dividing the total distance into two identical parts. To ensure the same electrode positioning in all conditions, all experimental testing was performed in one session.

### Subjects

Based on a previous study (Galpin et al., 2015), a sample size calculation was performed determining a power of 80% and allowing an effect size of 0.8 to be significant at the 5% level of significance. Based on the calculation a minimum of 15 subjects needed to be recruited to the study.

Sixteen men (age 22.8 ± 1.7 years, body mass 84.0 ± 8.7 kg, height 183.8 ± 6.7 cm) with 2.5 ± 1.5 years of free weights deadlift training experience volunteered for the study. All participants trained the deadlift on a weekly basis, but none were competing in weightlifting or powerlifting (2 RM: 148 ± 17.7 kg). The participants had to be over 18 years old, have a minimum of 1 year of weekly resistance training, perform the deadlift with proper technique and be free from injuries or pain that could reduce maximal effort during testing. They also had to refrain from resistance training 72 h before the testing. All volunteers were informed orally and in writing about the project, their anonymity and that they could not be identified in any way. All participants had to provide a written consent before being enrolled in the study. All appropriate consent pursuant to law was obtained before the start of the study.

### Procedures

Before the experimental session, three familiarization sessions were completed with 3 to 5 days separating them. In the first session the settings (i.e., preferred hand width and leg placement etc.) for each participant was defined in addition to familiarize with the execution of the three conditions. Also, in this session the length of the elastic bands in the top (resisted deadlift) or lower (assisted deadlift) position was adjusted so that the force output from the elastic bands was equated between the top position in the resisted deadlift and the lower position in the assisted deadlift for each participant (see Figure 1). Based on experience from pilot-testing, the elastic contribution (in the most stretched position) was set to ~50 percent of the 2 RM load. In the second familiarization session, the 2 RM load in each exercise was identified. The true 2 RM was defined as when the participants failed to complete the lift or were unable to lift the weights with proper technique, holding a straight/neutral back. In the third familiarization session, the routine from session two was repeated to replicate or adjust the 2 RM. The load was identified in one to three attempts. The free weight contributions to the 2 RM in the different conditions were: free-weights: 148 ± 18 kg, resisted deadlift: 115 ± 17 kg and assisted deadlift: 197 ± 18 kg.

Before each test session the same warm-up procedure was conducted. The procedure consisted of a 5 min general warm-up on a cycle ergometer followed by a specific warm-up performing the deadlift. The latter consisted of five repetitions at 40% of 2-RM, three repetitions at 60% of 2-RM, and two repetitions at 80% of 2-RM in each of the three conditions. A rest interval of 2–3 min was given between each set.

The lifting was performed in a power rack (Gym 2000, Modum, Norway) with a barbell (20 kg, Eleiko, Halmstad, Sweden), weight plates and elastic bands [dimension: 1 cm (width) × 0.5 cm (thickness), Ropes 302, Bungee, Norway]. The participants could use chalk, decide to lift with or without shoes and which grip to use, however, the same setting had to be used in all lifts. Furthermore, they were instructed to lift the barbell as fast as possible from the floor and until they reached a position where knees and hips were extended. Two assistants controlled the barbell after the completion of the ascending phase, due to the aim of the study and to reduce the amount of fatigue. Three to 5 min rest was given between each lift.

To identify the beginning and the end of the lift, as well as the different phases, a linear encoder was attached to the barbell (Ergotest Technology AS, Langesund Norway: sampling frequency of 200 Hz). The linear encoder was synchronized with the data acquisition system (MuscleLab 6000, Ergotest Technology AS, Langesund, Norway).

### Electromyography

Before placing the electrodes the skin was shaved, washed with alcohol and abraded in accordance to SENIAM recommendations (Hermens et al., 2000). Gel-coated, self-adhesive electrodes (11 mm contact diameter and a 2 cm center-to-center distance, Dri-Stick Silver circular surface EMG Electrodes AE-131, NeuroDyne Medical, USA) were placed in the presumed direction of the underlying muscle fibers on the dominant leg (defined as the one used to kick a ball). The electrode on gluteus maximus was placed at half the distance between the sacral vertebrae and the greater trochanter. For the semitendinosus the electrode was placed at 50% on the line between the ischial tuberosity and the medial epicondyle of the tibia. The biceps femoris was placed at 50% on the line between the ischial tuberosity and the lateral epicondyle of the tibia. The electrode on the erector spinae was located at L1, three centimeters lateral to the spinous process. The electrode on vastus lateralis was located two thirds down the line between spina iliaca anterior superior and the lateral side of the patella and finally, the electrode on vastus medialis was positioned four-fifths down the line between spina iliaca anterior superior and the cavity in front of the medial collateral ligament (www.seniam.org).

The root-mean-square (RMS) EMG obtained during the whole lift (lower and upper ascending phase) was used in the primary analysis (i.e., from the moment the barbell was lifted off the floor until the hip and knees were extended). The EMG signal was sampled at 1,000 Hz using a 16 bit A/D converter. To minimize noise from the surroundings, the raw EMG signal was amplified and filtered using a preamplifier located close to the sampling point. The preamplifier had a common mode rejection ratio of 106 dB, high cut frequency 500 Hz and low cut frequency 20 Hz (fourth-order Butterworth filter). Finally, the EMG signals were converted to RMS using a hardware circuit network (frequency response 450 kHz, averaging constant 12 ms, total error ± 0.5%). Commercial software (MuscleLab V10.4, Ergotest Technology AS, Langesund, Norway) was used to analyze the stored EMG data. To normalize the EMG values, maximal voluntary isometric contractions (MVCs) for all muscles were measured. The participants performed two attempts on each muscle and the order of the muscles was randomized. For the gluteus maximus, the participants lay in the prone position with a 90 degree angle in the knee (McGill and Marshall, 2012). The dominant leg performed manually resisted hip extensor MVCs. For the semitendinosus and biceps femoris, the participants, still lying in the prone position, performed knee flexor MVCs with a knee angle of ~45 degrees. For the erector spinae, resisted back extensor MVCs in the Biering-Sorenson position was performed (Zebis et al., 2013). For the vastii muscles the participants were seated in a chair with the knee and hip locked in a 90 degree angle. Each MVC lasted for ~5 s and the attempt with the highest 3 s amplitude being used in the analyses (McBride et al., 2006). A 2 min rest was given between each attempt. The ICC for electromyography has been reported to be high (0.90–0.95) as long the tests are performed in the same session (Lim and Sherwood, 2005).

### Kinetics

The participants were positioned on a force platform during all sessions (Ergotest Innovation A/S, Porsgrunn, Norway), which was calibrated in accordance with the manufacturer's specifications before testing. The sampling rate was 200 Hz. Average and peak force was calculated, based on the summed mass and acceleration of both bodyweight and barbell throughout the lift, using commercial software (MuscleLab v.10.4.37.4073, Ergotest Innovation A/S, Porsgrunn, Norway). The reliability of force platforms has been shown to be good (ICC 0.94) when measuring force (Cordova and Armstrong, 1996).

### Kinematics

A linear encoder was positioned directly under the barbell and used to calculate average and peak velocity, using the same software (MuscleLab v.10.4.37.4073, Ergotest Innovation A/S, Porsgrunn, Norway). The data from the linear encoder and force platform were synchronized which enabled the calculation of the time from the beginning of the lift to the peak force and the peak velocity. Using the linear encoder to measure velocity has been shown to be a highly reliable method (ICC 98%) (van den Tillaar and Ball, 2019).

### Statistical Analyses

The normality of the data was confirmed by visual inspection. Statistical analyses were performed with SPSS version 26 (SPSS, Inc., Chicago, IL, USA). Differences in neuromuscular activation, kinematics and kinetics were assessed using one-way repeated measures ANOVA with Bonferroni *post hoc* tests. The different conditions (free weights, assisted and resisted) were set as independent variables. All results are presented as mean ± 95% confidence interval (95% CI) and Cohen's d effect size (ES). An ES of 0.35 was considered small, 0.8 moderate and 1.5 large (Rhea, 2004). Statistical difference was accepted at *p* ≤ 0.05.

## Results

**Figure 2:**
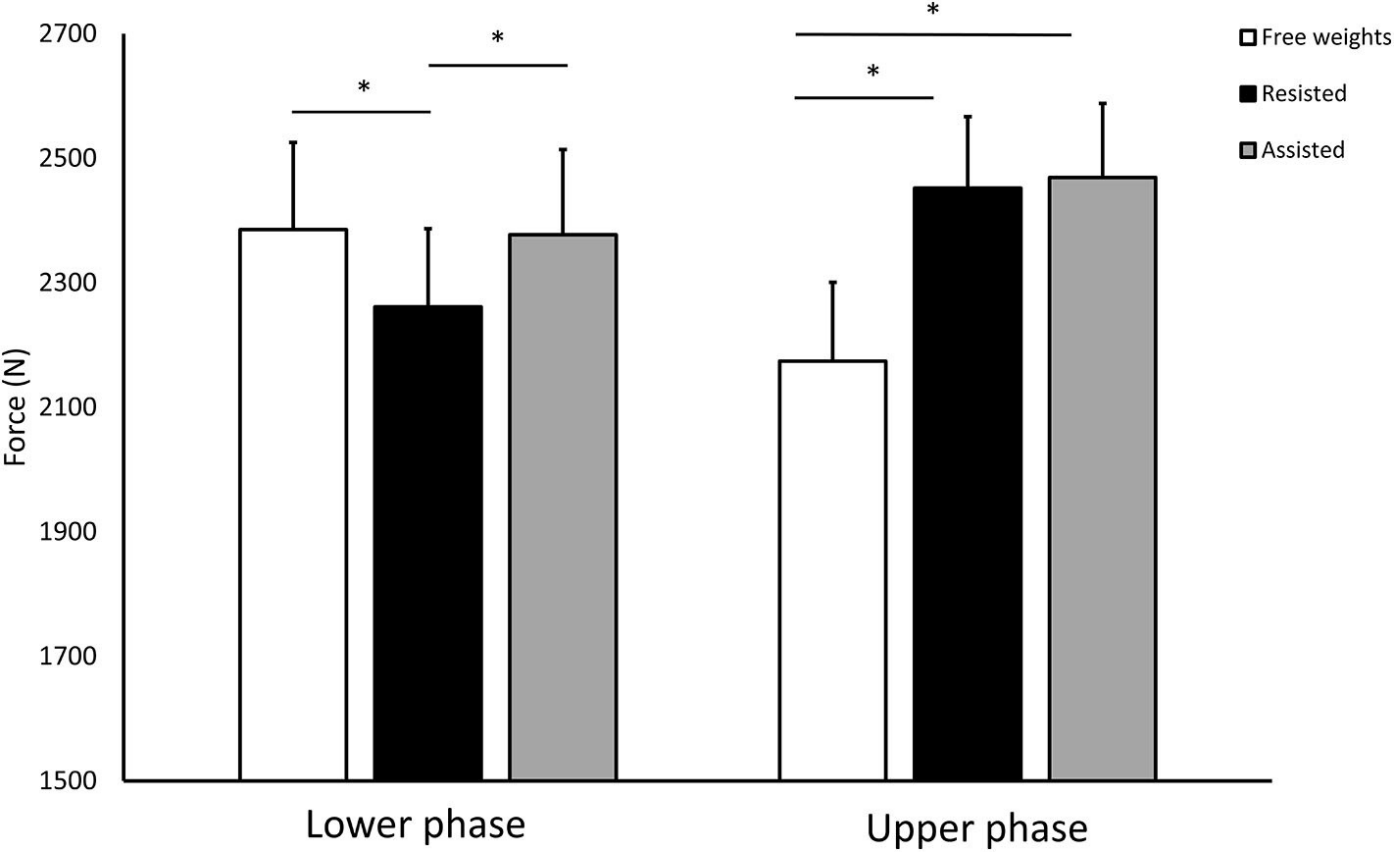
Average force output in the lower and upper phase during the free weights-, resisted-, and assisted deadlift. Values are means and 95% confidence interval. *p < 0.01.

Both the elastic band-conditions produced significantly higher average (resisted: *p* = 0.037, ES = 0.29; assisted: *p* < 0.001, ES = 0.58, Table 1) and peak (resisted: *p* = 0.002, ES = 0.59; assisted: *p* < 0.001. ES = 0.67) force output when compared to the free weight-condition. For the average force output, the same differences were found in the upper phase of the movement (resisted: *p* < 0.001, ES = 1.23; assisted: *p* < 0.001, ES = 1.28, Figure 2). In the lower phase, the free weight condition actually led to higher average force (*p* < 0.001, ES = 0.50) when compared to resisted deadlifts. There were no differences between the free weight and the assisted deadlift in the lower phase (*p* = 1.000). Comparing the two elastic band-conditions there were no differences for average or peak force output (*p* = 0.061–0.969). Time to peak force output were shorter for the free weights deadlift compared to the two elastic band-conditions (resisted: *p* < 0.001, ES = 1.58; assisted: *p* < 0.001, ES = 2.10).

**Table 1:** Force and barbell velocity during free weights-, resisted-, and assisted deadlift for the whole lift.

	**Free weights**	**Resisted**	**Assisted**
Average force (N)	2,298 (128)	2,366 (118)^a^	2,432 (120)^a^
Peak force (N)	2,525 (144)	2,673 (124)^a^	2,713 (150)^a^
Time to peak force (s)	0.56 (0.29)	1.57 (0.39)^a^	1.76 (0.31)^a^
Average velocity (m/s)	0.33 (0.05)	0.35 (0.06)	0.29 (0.05)
Peak velocity (m/s)	0.59 (0.10)	0.56 (0.10)	0.49 (0.07)^a^^b^
Time to peak velocity (s)	1.30 (0.26)	0.84 (0.23)^a^	0.94 (0.20)^a^

*Values are presented as mean and 95% confidence intervals*,

a *different from free weights (p ≤ 0.05)*,

b *different from resisted deadlift (p ≤ 0.05). N, newton; s, seconds; m/s, meter per second*.

There were no significant difference in the average velocity between the three conditions (*p* = 0.178–1.000). However, the assisted deadlift showed a lower peak velocity when compared to both free weights (*p* < 0.004, ES = 0.60) and resisted deadlift (*p* < 0.046, ES = 0.43) with no difference between the free weights and the resisted deadlift (*p* = 1.000). The time used to achieve peak velocity was higher in free weights compared to both resisted (*p* < 0.005, ES = 1.01) and assisted deadlifts (*p* = 0.010, ES = 0.83).

There were no significant differences in muscle activation for any of the muscles between the different conditions (*p* = 0.082–1.000, ES = 0.04–0.27, Table 2).

**Table 2:** Neuromuscular activation for the whole movement (% of MVC).

	**Free weights**	**Resisted**	**Assisted**
Gluteus maximus	110 (21)	112 (21)	113 (20)
Semitendinosus	85 (9)	90 (13)	90 (10)
Biceps femoris	116 (21)	122 (23)	121 (20)
Erector spinae	109 (29)	109 (21)	101 (19)
Vastus lateralis	141 (36)	138 (37)	123 (37)
Vastus medialis	155 (37)	163 (38)	145 (35)

*Values are presented as mean and 95% confidence intervals*.

## Discussion

The main finding of the present study was that resisted and assisted deadlifts led to higher average and peak force output when compared to the free weights deadlift. However, peak force was achieved faster when using free weights. Further, time to peak velocity was shorter for the elastic band conditions compared to free weights, but assisted deadlifts had a lower peak velocity compared to resisted and free weights deadlift.

In accordance with our hypothesis, the two elastic band-conditions produced higher force output throughout the ascending movement when compared to free weights. The difference between the assisted and resisted deadlift came close to statistically significant (*p* = 0.061). The rationale behind using elastic bands as a variable resistance is to maximize the load throughout the range of motion, especially in the upper parts of the lift where the external torque is reduced due to the reduction in moment arms. Also, in this part the external resistance is reduced since the barbell has acquired velocity (i.e., it is more difficult to accelerate it further). Our average force data strengthens this rationale showing the elastic band conditions to be superior to free weights only in the upper phase. In the lower phase the free weights produce more force than the resisted, but not than the assisted deadlifts. This is important to be aware of if exercises are selected toward improving the ability to produce force in the different parts of the movement. Our finding is likely explained by the fact that there is a curvilinear relationship between the lengthening of the bands and the tension in the band (McMaster et al., 2010). In the assisted deadlift the bands are stretched most in the lower part of the lift and therefore creating a potential to lift heavier loads in this phase when compared to the resisted deadlift where the bands are stretched the most in the upper parts of the lift.

The time to peak force was longer for the elastic band-conditions which could indicate that peak force occurs later (more upright position) in these conditions. This could also explain why the peak force was higher in the two elastic band-conditions, which agrees with our hypothesis. The leg extensors have a potential for creating a higher force output when the legs are more extended. For example, Bartolomei et al. (2019) found that the force output was higher during a mid-thigh pull compared to a mid-shin pull. Using the elastic bands increases the resistance as the barbell is lifted which, consequently would be more optimal for matching the muscles‘ ability to produce force throughout the lift.

In contrast to our hypothesis, there were no differences between the conditions in average velocity. Further, the assisted deadlift had a lower peak velocity compared to the free weights and the resisted deadlift. In both the free weights- and the resisted deadlift the force varies to a certain extent throughout the movement (~200 N between lower and upper phase). However, for the assisted deadlift the force appears to be more evenly distributed throughout the lift (92 N between lower and upper phase). The relationship between force and velocity is well-established and the differences in force variation could explain the differences in peak velocity.

The time to peak velocity was longer for the free weights compared to the two elastic band-conditions, which could be related back to the force pattern in the different conditions. In the free weights, the greatest force is achieved early in the lift and then reduced which would increase the acceleration and velocity throughout the lift. Combining the free weights with elastic bands would switch the pattern and direct the greatest force toward the end of the movement. Therefore, in these conditions, the barbell will accelerate most in the beginning of the lift and decelerate toward the top position.

Two previous studies have both compared force output, barbell velocity and muscle activation between free weights deadlift and resisted deadlifts using elastic bands (Galpin et al., 2015; Heelas et al., 2019). They also examined if different contributions from the elastic bands, i.e., using bands with more tension, affected the parameters. The findings from these studies differ from our results with both finding the resisted deadlifts to lead to higher average and peak velocity. Further Galpin et al. (2015) found the free weights to produce more average and peak force while Heelas et al. (2019) found the free weights-condition to activate several of the muscles more than compared to free weights and elastic bands. The differences are most likely a result of different approaches to the problem. Both Galpin et al. and Heelas et al. compared the conditions using the same absolute load, i.e., matching the total force in the upper position. One of the rationales behind variable resistance is to better match the muscles capability to produce torque throughout the movement. Therefore, we chose to compare the conditions using the same relative load, i.e., one rep at 2-RM which might have a better ecological validity toward training. Using the absolute load would scale down the load in the elastic band-conditions hence causing lower force output but greater velocity when compared to using the same relative load. This could also explain the decreased muscle activation in the elastic band-conditions as observed in the previous studies (Galpin et al., 2015; Heelas et al., 2019). Our force-data are supported by Swinton et al. (2011) who compared deadlift using free weights and free weights + chains. They found the chain-condition to be favorable regarding force output. This could be explained by the fact that their methodological approach is more similar to ours compared to Galpin et al. and Heelas et al. This paper matched the loading in the midpoint of the barbell trajectory, making the chains-condition heavier on top, but lighter in the bottom which resembles what happens when using relative intensity.

Surprisingly, and in contrast to our hypothesis, there were no differences in neuromuscular activation in any of the muscles between the three conditions. Previous studies have shown differences when comparing exercises performed with free weights in combination with elastic bands and free weights only when using the same relative load (Andersen et al., 2016, 2019). In both studies, the difference has only been apparent when a considerable amount of resistance comes from the elastic bands (mean 30–35% resistance). In the present study the elastic resistance was 50% of the 2-RM load, therefore the different findings was unexpected. However, in contrast to the previous studies, the execution of the exercises in our study was attempted to be performed explosively, which could increase the acceleration and hence compensate for differences in barbell load. Finally, it has been shown that muscle activation is relatively similar between 70 and 90% of 1-RM when the load is lifted with maximal intended velocity (van den Tillaar et al., 2019). Hence, differences in force may be too small to generate differences in muscle activation.

Some limitations need to be addressed. Only resistance-trained males were recruited to the study and the findings cannot necessarily be generalized to other populations. Further, none of the subjects were familiar with performing deadlifts in combination with elastic bands. Irrespective of three familiarization sessions, the results may have been different if the participants had more experience in using variable resistance. We did not analyze the eccentric phase, which could have affected the total movement. However, among resistance-trained subjects, the deadlift is often performed by lifting the barbell up before dropping to the floor. In this study, heavy loading was used, and it is possible that a submaximal loading would have changed the kinetics and muscle activation. However, since all the tests were performed in one session, which is recommended for acute EMG studies, we wanted to expose the participants to the minimal amount of fatigue as possible. Further, EMG only gives an estimate of the neuromuscular activation and there is a possibility for crosstalk from neighboring muscles (Farina et al., 2004). There are also additional methodological limitations when assessing EMG during dynamic muscle contractions (Farina, 2006). However, these limitations should be minimized as all data was collected in one session—removing potential error arising from replacing electrodes (Mathiassen et al., 1995).

The practical implications of the present study suggest that when comparing free weight deadlift and assisted and resisted deadlift, athletes and practitioners wanting to focus on producing as much force as possible when performing the deadlift, should combine free weights with elastic bands instead of free weights only. However, if force at a high degree of flexed knee and hips is the primary focus, free weights together with assisted deadlifts are the best alternatives. Further, assisted deadlifts seems to produce an evenly high force output through the whole movement, which could be beneficial for many sport movements, for example in scrummages in rugby and American football. However, resisted deadlifts together with free weights, seem to favor more explosive factors such as maximal velocity which is important in other movements such as sprint and jumping. Finally, if the training is directed toward movements requiring a high amount of force in the early phase and peak velocity in the later phase of the extension of the legs, then free weights seem more optimal, while the elastic band-conditions favor the opposite.

In conclusion, performing the assisted and resisted deadlift increased the average-, peak force, and time to peak force in addition to reduce the time to peak velocity when compared to free weights deadlift. The peak velocity was lower during the assisted deadlift compared to the free weights and resisted deadlift. Finally, there were no differences between the conditions in muscle activation.

## Data Availability Statement

The raw data supporting the conclusions of this article will be made available by the authors, without undue reservation.

## Ethics Statement

Ethical review and approval was not required for the study on human participants in accordance with the local legislation and institutional requirements. The patients/participants provided their written informed consent to participate in this study.

## Author Contributions

All authors listed have made a substantial, direct and intellectual contribution to the work, and approved it for publication.

## Conflict of Interest

The authors declare that the research was conducted in the absence of any commercial or financial relationships that could be construed as a potential conflict of interest.

## References

[B1] Andersen V. Fimland M. S. Kolnes M. K. Jensen S. Laume M. Saeterbakken A. H. (2016). Electromyographic comparison of squats using constant or variable resistance. J. Strength Cond. Res. 30, 3456–3463. 10.1519/JSC.0000000000001451 27100320

[B2] Andersen V. Fimland M. S. Mo D. A. Iversen V. M. Larsen T. M. Solheim F.. (2019). Electromyographic comparison of the barbell deadlift using constant versus variable resistance in healthy, trained men. PLoS ONE 14:e0211021. 10.1371/journal.pone.0211021 30668589 PMC6342300

[B3] Argus C. K. Gill N. D. Keogh J. W. Blazevich A. J. Hopkins W. G. (2011). Kinetic and training comparisons between assisted, resisted, and free countermovement jumps. J. Strength Cond. Res. 25, 2219–2227. 10.1519/JSC.0b013e3181f6b0f4 21654341

[B4] Bartolomei S. Rovai C. Lanzoni I. M. di Michele R. (2019). Relationships between muscle architecture, deadlift performance, and maximal isometric force produced at the midthigh and midshin pull in resistance-trained individuals. J. Strength Cond. Res. 10.1519/JSC.0000000000003455. [Epub ahead of print]. 31895282 PMC10842658

[B5] Cordova M. L. Armstrong C. W. (1996). Reliability of ground reaction forces during a vertical jump: implications for functional strength assessment. J. Athl. Train 31, 342–345. 16558421 PMC1318919

[B6] Farina D. (2006). Interpretation of the surface electromyogram in dynamic contractions. Exerc. Sport Sci. Rev. 34, 121–127. 10.1249/00003677-200607000-00006 16829739

[B7] Farina D. Merletti R. Enoka R. M. (2004). The extraction of neural strategies from the surface EMG. J. Appl. Physiol. 96, 1486–1495. 10.1152/japplphysiol.01070.2003 15016793

[B8] Frost D. M. Cronin J. Newton R. U. (2010). A biomechanical evaluation of resistance: fundamental concepts for training and sports performance. Sports Med. 40, 303–326. 10.2165/11319420-000000000-00000 20364875

[B9] Gabriel D. A. Kamen G. Frost G. (2006). Neural adaptations to resistive exercise: mechanisms and recommendations for training practices. Sports Med. 36, 133–149. 10.2165/00007256-200636020-00004 16464122

[B10] Galpin A. J. Malyszek K. K. Davis K. A. Record S. M. Brown L. E. Coburn J. W.. (2015). Acute effects of elastic bands on kinetic characteristics during the deadlift at moderate and heavy loads. J. Strength Cond. Res. 29, 3271–3278. 10.1519/JSC.0000000000000987 26079737

[B11] Heelas T. Theis N. Hughes J. D. (2019). Muscle activation patterns during variable resistance deadlift training with and without elastic bands. J. Strength Cond. Res. 10.1519/JSC.0000000000003272. [Epub ahead of print]. 31498223

[B12] Hermens H. J. Freriks B. Disselhorst-Klug C. Rau G. (2000). Development of recommendations for SEMG sensors and sensor placement procedures. J. Electromyogr. Kinesiol. 10, 361–374. 10.1016/S1050-6411(00)00027-4 11018445

[B13] Kompf J. Arandjelovic O. (2017). The sticking point in the bench press, the squat, and the deadlift: similarities and differences, and their significance for research and practice. Sports Med. 47, 631–640. 10.1007/s40279-016-0615-9 27600146 PMC5357260

[B14] Lim H. K. Sherwood A. M. (2005). Reliability of surface electromyographic measurements from subjects with spinal cord injury during voluntary motor tasks. J. Rehabil. Res. Dev. 42, 413–422. 10.1682/JRRD.2004.07.0079 16320138

[B15] Mathiassen S. E. Winkel J. Hagg G. M. (1995). Normalization of surface EMG amplitude from the upper trapezius muscle in ergonomic studies - a review. J. Electromyogr. Kinesiol. 5, 197–226. 10.1016/1050-6411(94)00014-X 20719652

[B16] McBride J. M. Cormie P. Deane R. (2006). Isometric squat force output and muscle activity in stable and unstable conditions. J. Strength Cond. Res. 20, 915–918. 10.1519/00124278-200611000-00031 17194253

[B17] McBride J. M. Larkin T. R. Dayne A. M. Haines T. L. Kirby T. J. (2010). Effect of absolute and relative loading on muscle activity during stable and unstable squatting. Int. J. Sports Physiol. Perform. 5, 177–183. 10.1123/ijspp.5.2.177 20625190

[B18] McGill S. M. Marshall L. W. (2012). Kettlebell swing, snatch, and bottoms-up carry: back and hip muscle activation, motion, and low back loads. J. Strength Cond. Res. 26, 16–27. 10.1519/JSC.0b013e31823a4063 21997449

[B19] McMaster D. T. Cronin J. McGuigan M. (2009). Forms of variable resistance training. Strength Cond. J. 31, 50–64. 10.1519/SSC.0b013e318195ad32

[B20] McMaster D. T. Cronin J. McGuigan M. R. (2010). Quantification of rubber and chain-based resistance modes. J. Strength Cond. Res. 24, 2056–2064. 10.1519/JSC.0b013e3181dc4200 20613648

[B21] Rhea M. R. (2004). Determining the magnitude of treatment effects in strength training research through the use of the effect size. J. Strength Cond. Res. 18, 918–920. 10.1519/00124278-200411000-00040 15574101

[B22] Shoepe T. C. Ramirez D. A. Almstedt H. C. (2010). Elastic band prediction equations for combined free-weight and elastic band bench presses and squats. J. Strength Cond. Res. 24, 195–200. 10.1519/JSC.0b013e318199d963 19816220

[B23] Swinton P. A. Stewart A. D. Keogh J. W. Agouris I. Lloyd R. (2011). Kinematic and kinetic analysis of maximal velocity deadlifts performed with and without the inclusion of chain resistance. J. Strength Cond. Res. 25, 3163–3174. 10.1519/JSC.0b013e318212e389 21993040

[B24] van den Tillaar R. Andersen V. Saeterbakken A. H. (2014). The existence of a sticking region in free weight squats. J. Hum. Kinet. 42, 63–71. 10.2478/hukin-2014-0061 25414740 PMC4234771

[B25] van den Tillaar R. Andersen V. Saeterbakken A. H. (2019). Comparison of muscle activation and kinematics during free-weight back squats with different loads. PLoS ONE 14:e0217044. 10.1371/journal.pone.0217044 31095625 PMC6521994

[B26] van den Tillaar R. Ball N. (2019). Validity and reliability of kinematics measured with PUSH band vs. linear encoder in bench press and push-ups. Sports 7:207. 10.3390/sports7090207 31509960 PMC6784224

[B27] Wallace B. J. Bergstrom H. C. Butterfield T. A. (2018). Muscular bases and mechanisms of variable resistance training efficacy. Int. J. Sports Sci. Coach. 13, 1177–1188. 10.1177/1747954118810240

[B28] Zebis M. K. Skotte J. Andersen C. H. Mortensen P. Petersen H. H. Viskaer T. C.. (2013). Kettlebell swing targets semitendinosus and supine leg curl targets biceps femoris: an EMG study with rehabilitation implications. Br. J. Sports Med. 47, 1192–1198. 10.1136/bjsports-2011-090281 22736206

